# Targeting ACLY efficiently inhibits SARS-CoV-2 replication

**DOI:** 10.7150/ijbs.72709

**Published:** 2022-07-11

**Authors:** Terrence Tsz-Tai Yuen, Jasper Fuk-Woo Chan, Bingpeng Yan, Cynthia Cheuk-Ying Shum, Yuanchen Liu, Huiping Shuai, Yuxin Hou, Xiner Huang, Bingjie Hu, Yue Chai, Chaemin Yoon, Tianrenzheng Zhu, Huan Liu, Jialu Shi, Jinjin Zhang, Jian-Piao Cai, Anna Jinxia Zhang, Jie Zhou, Feifei Yin, Shuofeng Yuan, Bao-Zhong Zhang, Hin Chu

**Affiliations:** 1 State Key Laboratory of Emerging Infectious Diseases, Department of Microbiology, and Carol Yu Centre for Infection, School of Clinical Medicine, Li Ka Shing Faculty of Medicine, The University of Hong Kong, Pokfulam, Hong Kong Special Administrative Region, People's Republic of China.; 2 Department of Clinical Microbiology and Infection Control, The University of Hong Kong-Shenzhen Hospital, Shenzhen, Guangdong, People's Republic of China.; 3 Centre for Virology, Vaccinology and Therapeutics, Hong Kong Science and Technology Park, Hong Kong Special Administrative Region, People's Republic of China.; 4 Department of Microbiology, Queen Mary Hospital, Pokfulam, Hong Kong Special Administrative Region, People's Republic of China.; 5 Guangzhou Laboratory, Guangdong Province, China.; 6 Academician Workstation of Hainan Province, Hainan Medical University-The University of Hong Kong Joint Laboratory of Tropical Infectious Diseases, Hainan Medical University, Haikou, Hainan, People's Republic of China and The University of Hong Kong, Pokfulam, Hong Kong Special Administrative Region, China.; 7 Key Laboratory of Tropical Translational Medicine of Ministry of Education, Hainan Medical University, Haikou, Hainan, China.; 8 Department of Pathogen Biology, Hainan Medical University, Haikou, Hainan, China.; 9 CAS Key Laboratory of Quantitative Engineering Biology, Shenzhen Institute of Synthetic Biology, Shenzhen Institutes of Advanced Technology, Chinese Academy of Sciences; Shenzhen, People's Republic of China.

**Keywords:** COVID-19, metabolomics, ACLY, SARS-CoV-2, Delta, Omicron

## Abstract

The Coronavirus Disease 2019 (COVID-19) pandemic caused by severe acute respiratory syndrome coronavirus 2 (SARS-CoV-2) is the biggest public health challenge the world has witnessed in the past decades. SARS-CoV-2 undergoes constant mutations and new variants of concerns (VOCs) with altered transmissibility, virulence, and/or susceptibility to vaccines and therapeutics continue to emerge. Detailed analysis of host factors involved in virus replication may help to identify novel treatment targets. In this study, we dissected the metabolome derived from COVID-19 patients to identify key host factors that are required for efficient SARS-CoV-2 replication. Through a series of metabolomic analyses, *in vitro*, and *in vivo* investigations, we identified ATP citrate lyase (ACLY) as a novel host factor required for efficient replication of SARS-CoV-2 wild-type and variants, including Omicron. ACLY should be further explored as a novel intervention target for COVID-19.

## Introduction

Severe acute respiratory syndrome coronavirus 2 (SARS-CoV-2), the causative agent of Coronavirus Disease 2019 (COVID-19), is a lineage B betacoronavirus that emerged in late 2019 1-3. As of 28 February 2022, the virus has caused more than 434 million cases, including nearly 6 million deaths globally 4. In the past two years, new SARS-CoV-2 variants with enhanced transmissibility, virulence, and/or immune-evasiveness have emerged. For instance, the recently emerged Omicron is a variant of concern (VOC) that is associated with reduced susceptibility to vaccine-induced neutralizing antibody response and higher transmissibility despite lower pathogenicity 5-8. This and other emerging VOCs pose severe challenges to the public health authorities in terms of pandemic control and treatment of SARS-CoV-2-infected patients 6, 9-11.

Metabolomics is the systemic profiling of chemical processes concerning metabolites 12. These metabolomes represent the metabolite profiles generated at the end of cellular processes and can provide an overview of the physiological state of the targets at a particular moment. In this study, we aim to dissect the metabolome of COVID-19 patients to identify key host factors that are required for efficient SARS-CoV-2 replication. The extracted metabolites from COVID-19 patients' plasma samples were analyzed with both gas chromatography-mass spectrometry (GC-MS)-based and liquid chromatography-mass spectrometry (LC-MS)-based metabolomics. By multiple steps of data validation, combination, and analysis, we found that the metabolites in the tricarboxylic acid (TCA) cycle were significantly perturbed in COVID-19 patients. Importantly, we identified two inhibitors that target ATP citrate lyase (ACLY) within the TCA cycle, SB 204990 and Bempedoic acid, that effectively inhibited SARS-CoV-2 replication.

## Materials and Methods

### Plasma collection and metabolites extraction

Plasma samples were collected from COVID-19 patients whose respiratory tract specimens tested positive for SARS-CoV-2 and blood donors who tested negative for SARS-CoV-2 by RT-qPCR. Blood was collected using potassium-EDTA blood collection tubes and plasma was separated by centrifugation at 2000 rpm for 10 min. Metabolites extraction for GC-MS was performed according to a previously described protocol with slight modifications 13, 14. Plasma (20 µL) was thawed on ice and 80 µL of chloroform/methanol (v/v 2:1) was added, followed by vortexing for 30s, leaving the samples on ice, and vortexing again for 30 s. The samples were then incubated for 5 min at 1500 rpm at 4 °C in the orbital mixer. After that, samples were centrifuged at 4500 rpm for 10 min at 4 °C. Both upper and bottom phases were transferred to centrifuge tubes and dried in a Labconco Centrivap cold trap concentrator for storage at -80 °C. Metabolites extraction for LC-MS was performed according to a previously described protocol 15. 20 µL of ice-cold methanol that contained internal standards and butylated hydroxytoluene (BHT) was first added to plasma (40 µL). Samples were vortexed for 5s and kept on ice. Then, 800 µL of acetonitrile/isopropanol/water (v/v/v 3:3:2) was added, followed by vortexing for 30 s and incubation for 5 min at 1500 rpm at 4 °C in the orbital mixer. After that, samples were centrifuged at 14000 rpm for 10 min at 4 °C. The supernatant was transferred to centrifuge tubes and was split into two aliquots (410 µL for negative mode and another 410 µL for positive mode). Finally, all aliquot samples were dried in a Labconco Centrivap cold trap concentrator for storage at -80 °C.

### GC-MS-based targeted metabolomics

For polar metabolites, GC-MS chromatogram was acquired in SCAN and MRM mode in an Agilent 7890B GC - Agilent 7010 Triple Quadrapole Mass Spectrometer system (Santa Clara, CA, USA). The sample was separated through an Agilent (Santa Clara, CA, USA) DB-5MS capillary column (30 m × 0.25 mm ID, 0.25 μm film thickness) under constant flow at 1 mL min^-1^. The GC oven program started at 50°C (hold time 1 minute) and temperature was first increased at a rate of 10°C min^-1^ to 120°C, then 3°C min^-1^ to 150°C, next 10°C min^-1^ to 200°C, and finally 30°C min^-1^ to 280°C (hold time 5 minutes). Inlet temperature and transfer line temperature were 250°C and 280°C respectively. Characteristic quantifier and qualifier transitions were monitored in MRM mode during the run. Mass spectra from m/z 50-500 were acquired in SCAN mode 16. For Non-polar fatty acids, GC/MS equipment was same as polar metabolites analysis. The sample was separated through an Agilent DB-23 capillary column (60 m × 0.25 mm ID, 0.15 μm film thickness) under constant pressure at 33.4 psi. The GC oven program started at 50°C (hold time 1 minute) and was increased to 175°C at a ramp rate of 25°C min^-1^. The temperature was then raised to 190°C (hold time 5 minutes) at a ramp rate of 3.5°C min^-1^. Finally, the temperature was raised to 220°C (hold time 4 minutes) at a ramp rate of 2°C min^-1^. Inlet temperature and transfer line temperature were 250°C and 280°C respectively. Characteristic fragment ions (m/z 55, 67, 69, 74, 79, 81, 83, 87, 91, 93, 95, 96, 97, 115, 127, 143) were monitored in SIM mode throughout the run. Mass spectra from m/z 50-350 were acquired in SCAN mode 17.

### LC-MS-based untargeted metabolomics

Ultra-performance liquid chromatography coupled to quadrupole time-of-flight mass spectrometer (UPLC-Q-TOF-MS) analytical platform (Waters Corp., Milford, MA, USA) was used to perform untargeted metabolomics for hydrophilic metabolites and polar lipids characterization. The chromatography was performed on a Waters Acquity UPLC BEH Amide column (150 × 2.1 mm; 1.7 μm), the mobile phases and gradient elution were the same as previously described 15. The mass spectrometer was operated in MS^E^ mode and the data was acquired in both positive and negative modes. Mass spectral data was acquired over the m/z range of 100 to 1000. Collision energy was applied at the range from 20 to 40 eV for fragmentation to allow putative identification and structural elucidation of metabolites. Exogenous metabolite standards were applied for sample preparation and LC-MS analysis for monitoring the metabolites coverage and extraction efficiency. A total of 7 lipid internal standards were applied for sample preparation and LC-MS analysis for monitoring extraction efficiency including Succinic acid-d6-ISTD, L-Leucine-d_10_-ISTD, Salicylic acid-d_4_-ISTD, L-GLUTAMINE-d_5_-ISTD, Creatine-d_3_-ISTD, L-arginine-^15^N2-ISTD, and Trimethylamine N-oxide-d_9_-ISTD. Commercial standards were used for metabolites identification. They were purchased from Cambridge isotope lab (Andover, MA, USA) and Cayman Chemical (Ann Arbor, MI, USA). Additionally, QC samples were injected at the beginning of the run and after every 6 or 8 samples for monitoring the system variation. QC samples were pooled and prepared by mixing equal aliquots for all the biological samples 18, 19.

### Data processing, statistical analysis and metabolites identification in untargeted metabolomic

Untargeted metabolomics study data was processed to a usable data matrix by the MS-DIAL software for further statistical analysis 15, 20. MetaboAnalyst 4.0 (http://www.metaboanalyst.ca) and SIMCA-P V12.0 (Umetrics, Umeå, Sweden) were used for univariate and multivariate analysis, respectively. Prior to statistical analysis, the data matrix was performed QC or DNA-based normalization for better comparison 21. In the univariate analysis, only the FDR adjusted p-value that is less than 0.05 and fold change that is more than 1.5 or less than 0.67 were used as the criteria for selecting significant metabolites. In multivariate analysis, the metabolite features were first subjected to Pareto scaling, and followed by partial least squares-discriminant analysis (PLS-DA) to find important variables with discriminative power. PLS-DA model was evaluated with the relevant R2 and Q2. The Variable Importance in Projection (VIP), which reflects both the loading weights for each component and the variability of the response explained by this component, was used to select the metabolites 22. The significant metabolites were identified by searching accurate MS and MS/MS fragmentation pattern data in the MS-DIAL database 20, MassBank of North America (MoNA, http://mona.fiehnlab.ucdavis.edu/) and METLIN database (http://metlin.scripps.edu/). For confirmation of metabolite identities using authentic chemical standards, the MS/MS fragmentation patterns of the chemical standards were compared with those of the candidate lipids measured under the same LC-MS condition. Pathway analysis was performed by MetaboAnalyst and KEGG mapper 23.

### Viruses and biosafety

SARS-CoV-2 wild-type (WT, HKU-001a, GenBank: MT230904), B.1.617.2 (Delta, GenBank: OM212471), and B.1.1.529.1 (Omicron BA.1, GenBank: OM212472) viruses were available at the Department of Microbiology of The University of Hong Kong (HKU). They were all isolated from laboratory-confirmed COVID-19 patients in Hong Kong 24, 25. VeroE6-TMPRSS2 cells were used to culture and titrate virus stocks, and the sequences of the viruses were confirmed with nanopore sequencing. All the *in vivo* and *in vitro* experiments with live SARS-CoV-2 were performed according to the approved standard operating procedures of our Biosafety Level 3 facility 26, 27.

### Cell culture

The cell lines used in this study were available in our laboratory as previously described 24. Caco2 cell was acquired from ATCC (ATCC HTB-37) and cultured in Dulbecco's modified Eagle's medium (DMEM) (Gibco, Amarillo, Texas, USA) according to supplier's manual and guidance. VeroE6-TMPRSS2 cell was acquired from the Japanese Collection of Research Bioresources (JCRB) Cell Bank (JCRB1819) and cultured in Dulbecco's modified Eagle's medium (DMEM) (Gibco, Amarillo, Texas, USA) according to instructions. All cell lines that were used in this study underwent mycoplasma testing regularly and were cultivated in mycoplasma-free environment.

### RNA extraction and qRT-PCR

RNA was extracted from infected Caco2 cells using the QIAsymphony RNA kit (Qiagen, Germany), and RNA was extracted from hamster lung tissues using the RNeasy Mini kit (Qiagen, Germany). Viral gene copies of SARS-CoV-2 was quantified by the RNA-dependent RNA polymerase (RdRp) using the QuantiNova Probe RT-PCR kit (Qiagen, Germany) as previously described 28, 29.

### Cell viability assay (CC_50_)

Cell viability was quantified by CellTiter-Glo luminescent cell viability assay kit (Promega, USA) as we previously described 30. Caco2 cells were treated with selected inhibitors at a series of concentration (0-100µM) for 24 hours, and followed by manufacturer's instructions to detect luminescent signal using the Victor X3 2030 Multilabel reader (Perkin Elmer, USA).

### IC_50_ of the chosen inhibitors

Caco2 cells were challenged with 0.1 multiplicity of infection (MOI) from one of the three SARS-CoV-2 (WT, Delta, and Omicron) strains. At 2 hours post-infection, the virus inoculum was removed, cells were washed with phosphate-buffered saline (PBS) for 3 times, and they were treated with the chosen inhibitors at a titration of different concentrations (0-100 µM). At 24 hours post-infection, supernatant was harvested, followed by both RNA extraction and qRT-PCR to quantify for RdRp gene copies. IC_50_ was then calculated using GraphPad Prism 6 as previously mentioned 31, 32.

### Small interfering RNA (siRNA) knockdown

SMARTPool ON-TARGETplus human ACLY siRNA was purchased from Dharmacon, and Caco2 cells were used to perform the transfection of siRNA as previously described with minor modifications 33, 34. Cells were seeded on day 1 and 70nM of ACLY or nontargeting (scrambled) siRNA were transfected into the cells with RNAiMAX and Opti-MEM on day 2. At 24 hours post-transfection, the cells were challenged by WT SARS-CoV-2 at 0.1 MOI. At 2 hours post-virus challenge, virus inoculum was removed, and the cells were washed 3 times with PBS. At 24 hours post-infection, supernatant and cell lysate were harvested, followed by both RNA extraction and qRT-PCR to quantify for viral RdRp and host ACLY gene copies.

### *In vivo* virus challenge in hamsters

The animal experiments were approved by the HKU Committee on the Use of Live Animals in Teaching and Research (CULATR). Briefly, 6-8 weeks old male and female Syrian hamsters were obtained from the Chinese University of Hong Kong Laboratory Animal Service Centre through the HKU Centre for Comparative Medicine Research (CCMR) 35, 36. The hamsters were kept with 65% humidity and 21-23^ o^C ambient temperature until virus challenge 37. Standard pellet food and water were given, and 12-hours-interval day/night cycle was provided for housing and husbandry. The hamsters were intranasally inoculated with 50 µL per hamster of SARS-CoV-2 WT or Omicron under anaesthesia with intraperitoneal ketamine (200 mg/kg) and xylazine (10 mg/kg) 38, 39. PBS was used to dilute SARS-CoV-2 WT and Omicron stocks to the concentration of 3×10^3^ PFU per hamster. At 6 hours post-infection each hamster was treated intraperitoneally with 10mg/kg of SB 204990 or 5% DMSO at a final volume of 1000 µL PBS. The infected hamsters were subsequently treated with SB 204990 or DMSO at 1, 2, and 3 days post infection for a total of 2 or 4 doses. All hamsters were sacrificed on day 2 or day 4 post-infection for virological and histological assessments.

### Infectious viral titer by plaque assay

Plaque assay was performed as we described previously 40, 41. Briefly, hamster lung tissues were harvested and homogenized in DMEM using the Tissue Lyzer II (Qiagen, Germany). Homogenized lung tissues were centrifuged down in full speed for 5 minutes. Supernatants were collected, and were 10-fold serially diluted to inoculate VeroE6-TMPRSS2 cells. At 2 hours post-infection, virus inoculum was removed, and the cells were washed with PBS for 3 times. 1:1 of 2% low-melting agarose: DMEM with 2% FBS and 1% P/S was then added to the cells. At 72 hours post-infection, the cells were fixed by 4% (wt/vol) paraformaldehyde for 24 hours. Fixed VeroE6-TMPRSS2 cells were stained with 0.5% crystal violet in 25% ethanol for viral titer determination.

### Immunofluorescence staining

Immunofluorescence staining was performed as we previously described with slight modifications 42-44. Briefly, the hamster lung tissues were first harvested and fixed using 4% (wt/vol) paraformaldehyde at room temperature for 24 hours before proceeding to paraffin-embedding and sectioning. The slides with lung tissues were then dewaxed, dehydrated, and antigen retrieved. Sudan Black B and 1% BSA were used to reduce autofluorescence and for blocking respectively, followed by overnight incubation with in-house rabbit polyclonal biotinylated anti-SARS-CoV-2 nucleocapsid protein antibody (1:4000). On the next day, the tissue slides were incubated with the donkey anti-rabbit FITC secondary antibody from ThermoFisher and were mounted with a DAPI mounting medium (Vector) to detect viral antigen. All fluorescence images were captured using the Olympus BX53 fluorescent microscope.

### Statistical analyses

All data that is presented in this study represented mean and standard deviations from at least three independent experiments. One-way analysis of variance (ANOVA) or student's t test were performed between three or more experimental groups and between two experimental groups respectively. All presented data was considered statistically significant only when *P* < 0.05.

## Results

### GC-MS-based identification of metabolome perturbations in COVID-19 patients

**Figure 1:**
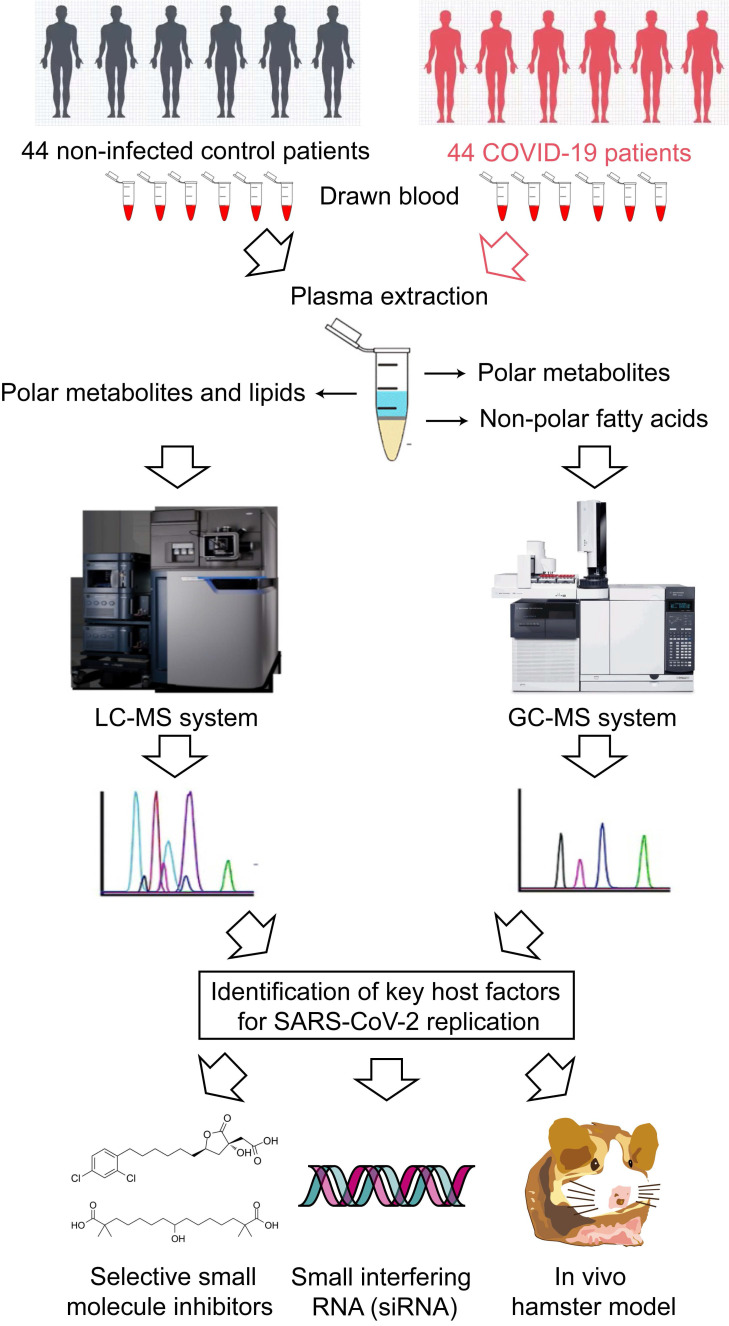
Schematic illustration of the study.

**Figure 2:**
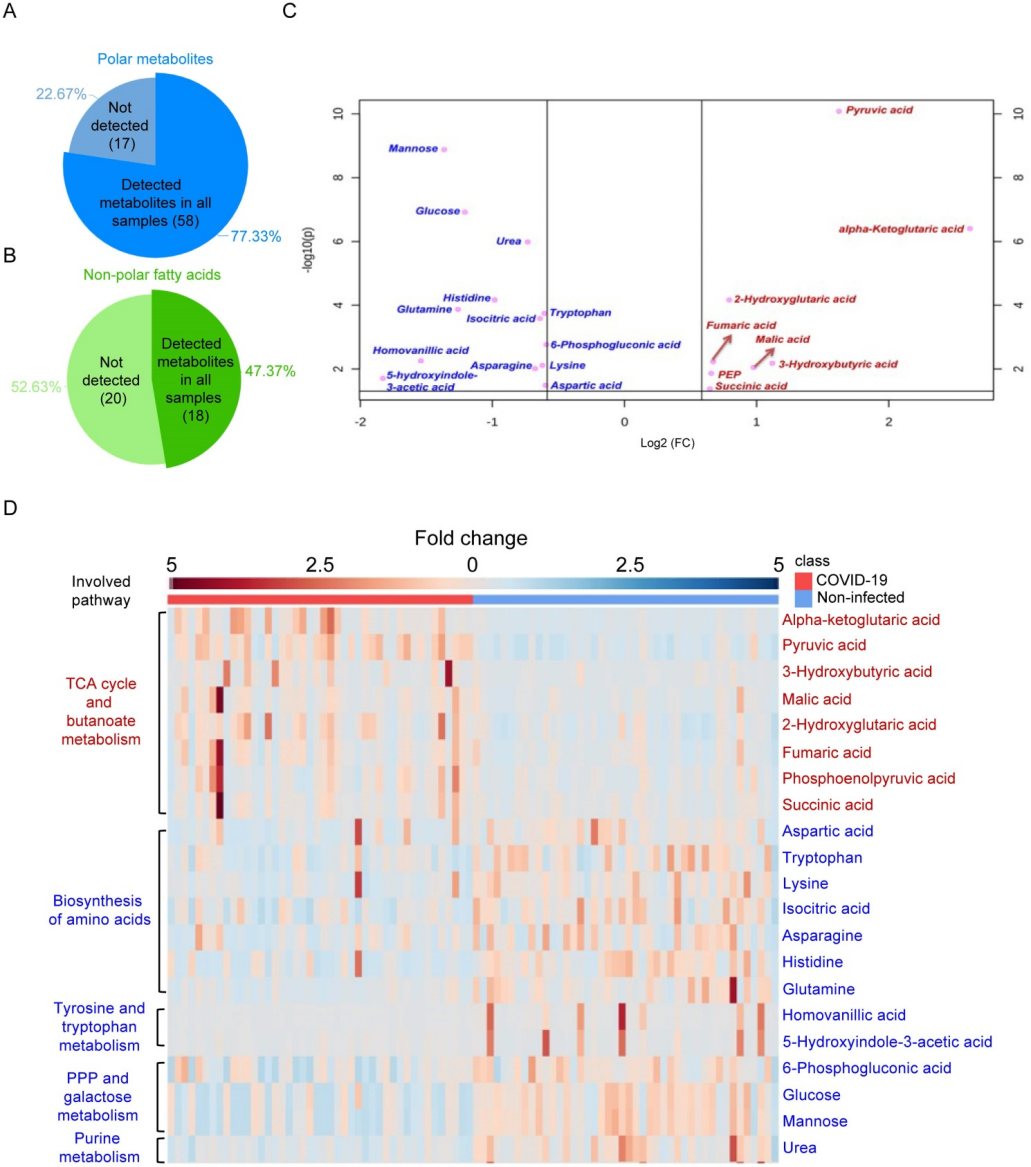
GS-MS-based targeted metabolomics was used to identify metabolites in COVID-19 patient plasma samples. (A) Pie chart showing the ratio of detected and non-detected polar metabolites using the GC-MS-based targeted metabolites. (B) Pie chart showing the ratio of detected and non-detected non-polar fatty acids using the GC-MS-based targeted metabolites. (C) Overview of the upregulated and downregulated metabolites in COVID-19 patients' plasma. (D) Heatmap showing the upregulated metabolites and their belonged pathways in both COVID-19 patients and non-infected donors.

A total of 44 laboratory-confirmed COVID-19 patients and 44 non-infected control patients were recruited for both GC-MS-based and LC-MS-based metabolomics (**Figure 1**). There were 24 males and 20 females in each group. The median age was 58 and 57.5 years for COVID-19 patients and non-infected control patients, respectively. A total of 78 polar metabolites and 38 non-polar fatty acids were analyzed by GC-MS-based targeted metabolomics, and they were normalized by corresponding internal standards and peak areas. Among them, 58 polar metabolites (**Figure 2A**) and 18 non-polar fatty acids (**Figure 2B**) could be detected. Further analysis of these 76 metabolites showed that 21 of them were significantly different between the plasma samples of COVID-19 patients and those of the non-infected controls (**Table 1**). We then compared the direction, magnitude, and statistical significance of these 21 metabolites. Our data revealed that 8 of these metabolites (mainly organic acids) were significantly upregulated, with the alpha-ketoglutaric acid exhibiting the largest fold change (around 6 folds) in COVID-19 patient plasma samples (**Figure 2C**). For the 13 downregulated metabolites, they belong to multiple classes including amino acids, organic acids, carbohydrates, and other metabolites (**Figure 2C**). Interestingly, 75% (6/8) of the upregulated metabolites, including alpha-ketoglutaric acid, pyruvic acid, malic acid, fumaric acid, phosphoenolpyruvic acid, and succinic acid are involved in the TCA cycle (**Figure 2D**). On the other hand, 46% (6/13) of the downregulated metabolites including aspartic acid, tryptophan, lysine, asparagine, histidine, and glutamine are involved in the biosynthesis of amino acids (**Figure 2D**). Taken together, our results indicated that SARS-CoV-2 infection activates the TCA cycle and exhausts the amino acid biosynthesis pathways.

### LC-MS-based untargeted metabolomics identified perturbed metabolites in COVID-19 patients

**Figure 3:**
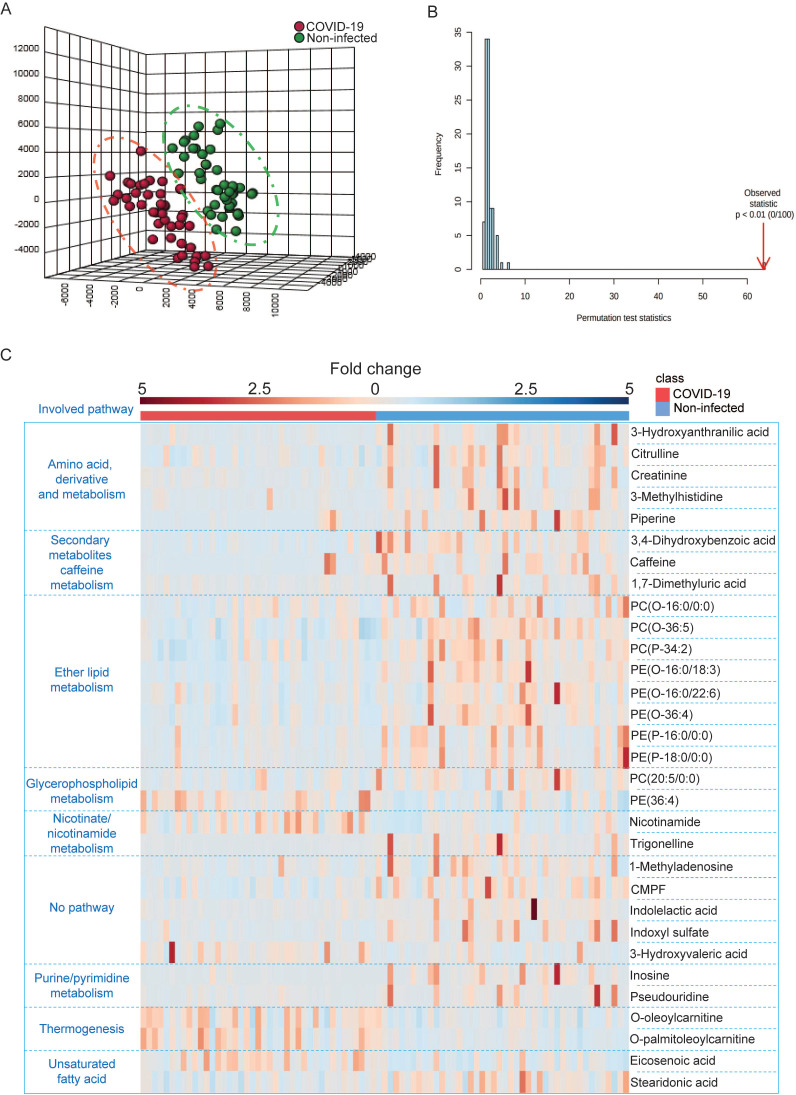
LC-MS-based untargeted metabolomics was used to identify perturbed metabolites in COVID-19 patient plasma samples. (A) PLS-DA model showing the metabolic profiles between the COVID-19 patients and non-infected controls were different. (B) Permutation test showing a statistically significant separation between the COVID-19 patients and non-infected donors. (C) Heatmap showing the upregulated metabolites and their belonged pathways in both COVID-19 patients and non-infected donors.

Next, we utilized LC-MS-based untargeted metabolomics for sample analysis and detected a total of 495 metabolic features in positive mode and 1354 metabolic features in negative mode, and the coefficient of variation (CV) values of all MS features in quality control (QC) samples were calculated. Our results indicated that 98.79% of all features in positive mode and 95.42% of all features in negative mode had CV values lower than 30%, which indicated stable data acquisition (**Table 2**). The metabolic profiles between COVID-19 patient and non-infected control patient plasma samples were clearly separated (**Figure 3A**), which exhibited metabolic features that contributed to the discrimination pattern between the two groups under PLS-DA pattern recognition. Since the PLS-DA model may overfit data, we further validated the model with the permutation test, which showed that the separation between the two groups was statistically significant (**Figure 3B**). Using LC-MS, we identified a total of 31 metabolites, including 25 downregulated and 6 upregulated metabolites. Their fold changes and involved pathways were visualized by heatmap (**Figure 3C**).

Notably, around 32% (10/31) of metabolites belonging to the polar lipid class that are involved in ether lipid/glycerophospholipid metabolism had a trend of downregulation. This suggested that lipids metabolism was perturbed in COVID-19 patient plasma samples. Consistent with the GC-MS-based targeted metabolomics, amino acids and their derivates exhibited declining trends in COVID-19 patient plasma samples. On the other hand, some of the upregulated metabolites belong to the carnitine class that is involved in thermogenesis. Collectively, the untargeted metabolomics indicated that multiple metabolic pathways were perturbed after SARS-CoV-2 infection.

### Global metabolic pathway analysis and receiver operating characteristic (ROC) curve analysis

**Figure 4:**
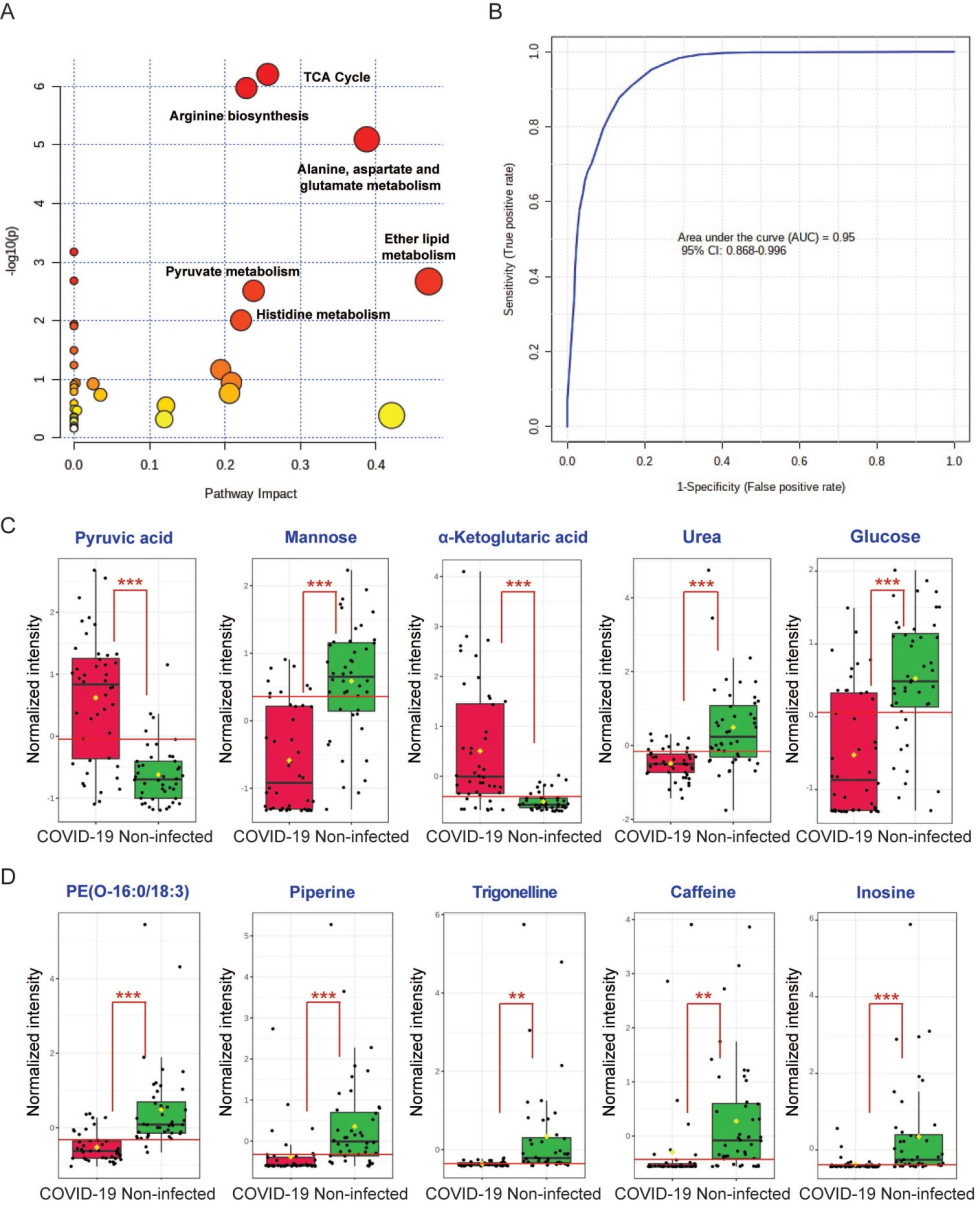
Combing all the identified metabolites from GC-MS and LC-MS. (A) Overview of the global metabolic pathway analysis from identified metabolites. The y-axis indicating the log10 transformed p-value after enrichment analysis, and the x-axis representing the pathway impact value calculated from the pathway topology analysis. (B) Receiver operating characteristic (ROC) curve analysis from the GC-MS and LC-MS platforms that were used in this study. (C) The top 5 perturbed metabolites from the GC-MS platform. (D) The top 5 perturbed metabolites from the LC-MS platform.

To comprehensively characterize the perturbed metabolic pathways in COVID-19 patient plasma samples, we combined the 52 significantly changed metabolites identified from the GC-MS and LC-MS platforms. In terms of the hit rate (hit metabolites/total metabolites in the pathway), the TCA cycle (7/20), arginine biosynthesis (6/14), and metabolism of alanine, aspartate and glutamate (7/28) were the top three perturbed pathways (**Figure 4A**). While ether lipid metabolism exhibited the largest pathway impact value, it was a lipid-independent pathway that did not belong to our targeted metabolic class. Next, we performed ROC curve analysis to assess the diagnostic value of metabolic biomarkers based on all the identified metabolites from the GC-MS and LC-MS platforms. The top 5 metabolites ranked by area under ROC curve (AUROC) were selected to generate the ROC curve plots. The representative ROC curve exhibited excellent discriminate capacity, which indicated that our model could differentiate plasma samples of COVID-19 patient from non-infected control using 5 of the identified metabolites (**Figure 4B**). The top 5 metabolites from the GC-MS platform included pyruvic acid and α-ketoglutaric acid (TCA cycle), mannose and glucose (galactose metabolism), and urea (purine metabolism) (**Figure 4C**). The top 5 metabolites from the LC-MS platform consisted of PE(O-16:0/18:3), piperine, trigonelline, caffeine, and inosine (**Figure 4D**). Interestingly, among these 10 metabolites, 8 were downregulated and only pyruvic acid and α-ketoglutaric acid (TCA cycle) were upregulated in COVID-19 patient plasma samples (**Figure 4C**). Taken together, by combining the metabolic pathway and ROC curve analysis, our data suggested that the TCA cycle is the most perturbed pathway in COVID-19 patient plasma samples.

### The three mini pathways within the TCA cycle are critical for SARS-CoV-2 infection

**Figure 5:**
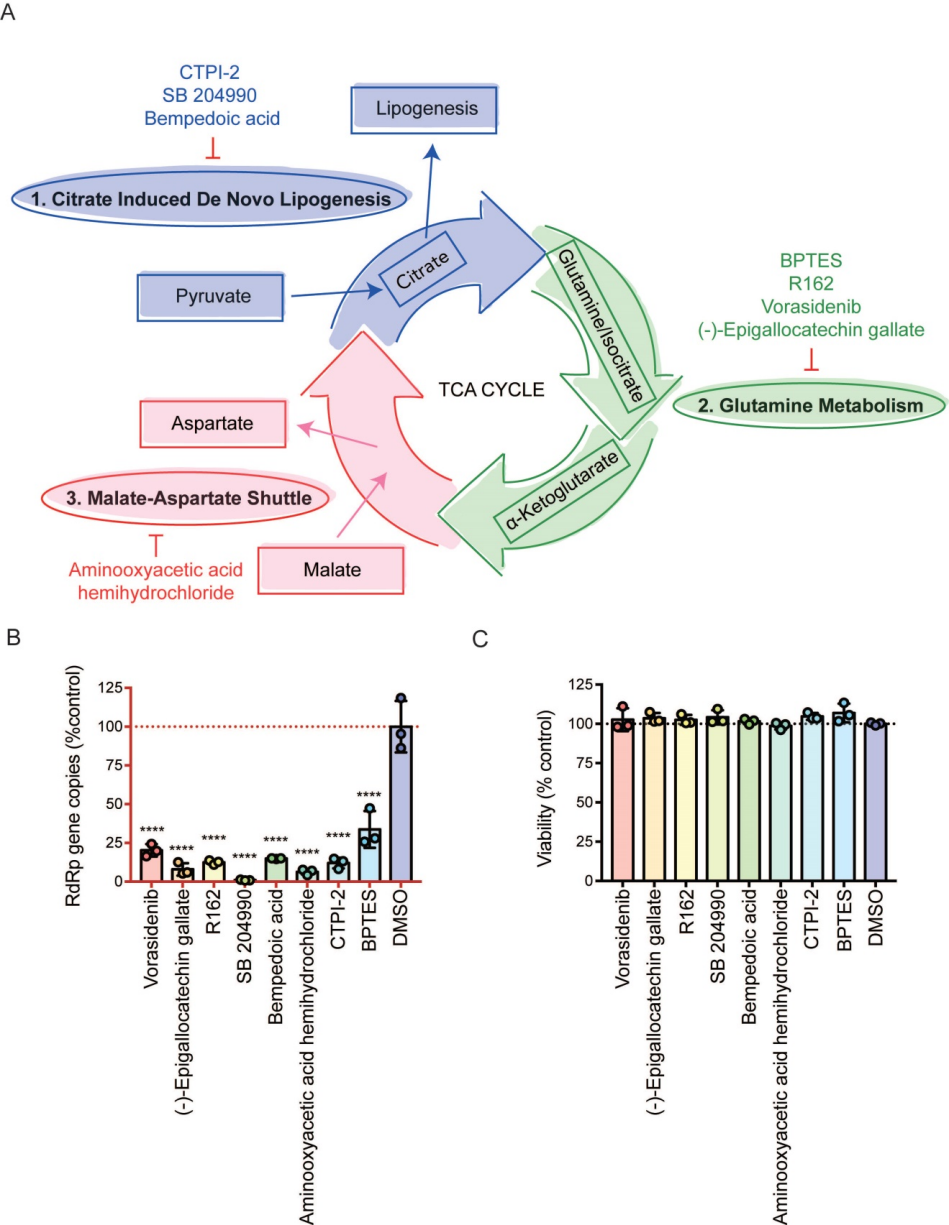
Inhibition of the three mini pathways within the TCA cycle reduced SARS-CoV-2 replication. (A) Schematic illustration of the three mini pathways including citrate induced *de novo* lipogenesis, glutamine metabolism, and malate-aspartate shuttle. (B) Caco2 cells were infected with WT SARS-CoV-2 at 0.1 MOI and treated with selective small molecule inhibitors including CTPI-2, SB 204990, Bempedoic acid, BPTES, R162, Vorasidenib, (-)-Epigallocatechin gallate, and aminooxyacetic acid hemihydrochloride at 50 µM. Supernatants were harvested at 24 hours post-infection. (C) The viabilities of all tested inhibitors in Caco2 cells without virus infection at 24 hours post-treatment. Data represented mean and standard deviation from three independent experiments. Statistical analyses in B were performed with one-way ANOVA, the differences were considered significant only when *P* < 0.05. *P < 0.05, **P < 0.01, ***P < 0.001, and ****P < 0.0001.

Viruses utilize host glycolysis as a source of ATP by inducing the TCA cycle to acquire an extensive amount of energy required during the viral replication cycle 45. From our metabolomics data, we identified several metabolites and pathways that may play important roles in SARS-CoV-2 infection. Based on their functions, we divided the complicated TCA cycle into three mini pathways for more detailed examinations, including (i) citrate induced *de novo* lipogenesis, (ii) glutamine metabolism, and (iii) malate-aspartate shuttle (**Figure 5A**).

The citrate induced *de novo* lipogenesis mini pathway is responsible for the conversion of acetyl-CoA from citrate by ACLY, the glutamine metabolism mini pathway is responsible for the conversion of α-ketoglutarate from glutamine by glutamate dehydrogenase and from isocitrate by isocitrate dehydrogenase, and the malate-aspartate shuttle is responsible for the translocation of electrons between the semipermeable inner membrane of mitochondrion. To investigate the mechanism between SARS-CoV-2 replication and these three mini pathways, we chose 8 different selective small molecule inhibitors that can specifically inhibit different enzymes within the three mini pathways. CTPI-2, SB 204990, and Bempedoic acid were selected to inhibit the citrate induced *de novo* lipogenesis mini pathway, BPTES, R162, Vorasidenib, and (-)-Epigallocatechin gallate were selected to inhibit the glutamine metabolism mini pathway, and aminooxyacetic acid hemihydrochloride was selected to inhibit the malate-aspartate shuttle mini pathway. Caco2 cell was selected to perform the *in vitro* experiments as it is a well-established cell model with robust SARS-CoV-2 replication, and supports SARS-CoV-2 entry through both plasma membrane and endosomal entry pathways 24. SARS-CoV-2-infected cells were treated with the 8 chosen inhibitors at a final concentration of 50 µM for 24 hours. Our results suggested that all 8 inhibitors significantly reduced SARS-CoV-2 replication (**Figure 5B**) at nontoxic concentrations (**Figure 5C**). At 50 µM, the citrate induced *de novo* lipogenesis mini pathway inhibitors reduced viral gene copies by 85.1 to 99%, while the glutamine metabolism mini pathway inhibitors reduced viral gene copies by 66.3 to 92.1%, and the malate-aspartate shuttle mini pathway inhibitor reduced viral gene copies by 93.7% (**Figure 5B**).

### Targeting the citrate induced *de novo* lipogenesis mini pathway potently inhibits SARS-CoV-2 replication

**Figure 6:**
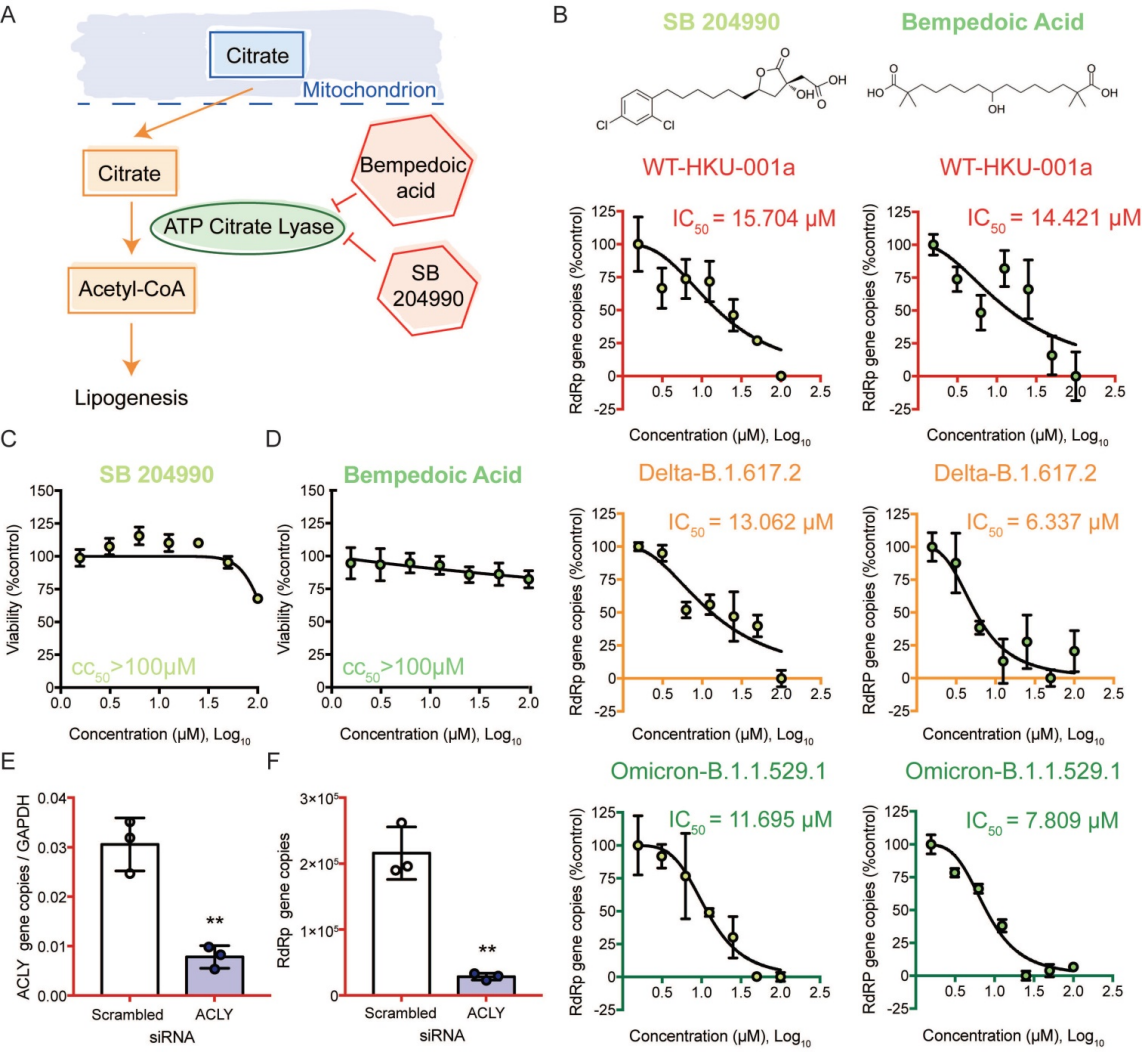
ACLY inhibition efficiently reduced the replication of SARS-CoV-2 wildtype and variants. (A) Schematic illustration of the citrate induced *de novo* lipogenesis mini pathway. (B) Caco2 cells were infected with SARS-CoV-2 WT, Delta, or Omicron BA.1 at 0.1 MOI and treated with SB 204990 or Bempedoic acid at a titration of different concentration (100, 50, 25, 12.5, 6.25, 3.125, or 1.5625 µM) or were treated with DMSO. Supernatants were harvested at 24 hours post-infection. The IC_50_ of SB 204990 and Bempedoic acid for SARS-CoV-2 WT, Delta, or Omicron BA.1 were calculated. (C and D) The CC_50_ of SB 204990 and Bempedoic acid in Caco2 cells were determined without virus infection at day 1. (E and F) Caco2 cells were transfected with ACLY or scrambled siRNA and infected with WT SARS-CoV-2 at 0.1 MOI. (E) The knockdown efficiency of ACLY is evaluated with qPCR. (F) Supernatants from SARS-CoV-2-infected cells treated with ACLY or scrambled siRNA were harvested at 24 hours post-infection. Virus replication was quantified with qPCR analysis. The IC_50_ and CC_50_ values in (B and D) were calculated with GraphPad Prism 7. Data represented mean and standard deviation from three independent experiments. Statistical differences were considered significant when *P* < 0.05. **P < 0.01.

We then selected the citrate induced *de novo* lipogenesis mini pathway for further investigation since inhibitors that targeted this mini pathway showed the most potent antiviral effect against SARS-CoV-2 (**Figure 6A**). Citrate, a crucial metabolite in the TCA cycle, is first exported from the mitochondria through transporters. Once it is transported out of the mitochondria, citrate is then converted to acetyl-CoA by ACLY 46. This is a key step in fatty acid biosynthesis to generate acetyl-CoA for important biosynthetic pathways including lipogenesis and cholesterogenesis 46. To investigate the role of ACLY on SARS-CoV-2 replication, we treated SARS-CoV-2 WT-, Delta-, or Omicron-infected Caco2 cells with SB 204990 or Bempedoic acid (specific inhibitors of ACLY), and determined their half maximal inhibitory concentrations (IC_50_) values. Our results indicated that both SB 204990 and Bempedoic acid efficiently suppressed the replication of SARS-CoV-2 WT, Delta, and Omicron (**Figure 6B**). The IC_50_ of SB 204990 against SARS-CoV-2 WT, Delta, and Omicron was 15.7 µM, 13.1 µM, and 11.7 µM, respectively, while the IC_50_ of Bempedoic acid against SARS-CoV-2 WT, Delta, and Omicron was 14.4 µM, 6.3 µM, and 7.8 µM, respectively (**Figure 6B**). The reduction in viral gene copies was not due to cell death as the 50% cytotoxic concentrations (CC_50_s) of these two inhibitors in Caco2 cells were above 100µM (**Figures 6C and 6D**). To further verify our findings from the selective small molecule inhibitors that the host ACLY is exploited for efficient SARS-CoV-2 replication, we performed gene depletion with ACLY siRNA. ACLY siRNA effectively depleted ACLY expression in Caco2 cells with a knockdown efficiency of 74.5% (*P* = 0.0025) (**Figure 6E**). Importantly, in ACLY-depleted Caco2 cells, SARS-CoV-2 replication was reduced by 86.8% (*P* = 0.0013) when compared to the control group (scrambled siRNA treated) at 24 hours post-infection (**Figure 6F**). Collectively, these results indicated that ACLY is required for efficient SARS-CoV-2 replication.

### The selective ACLY inhibitor SB 204990 potently limits SARS-CoV-2 replication *in vivo*

**Figure 7:**
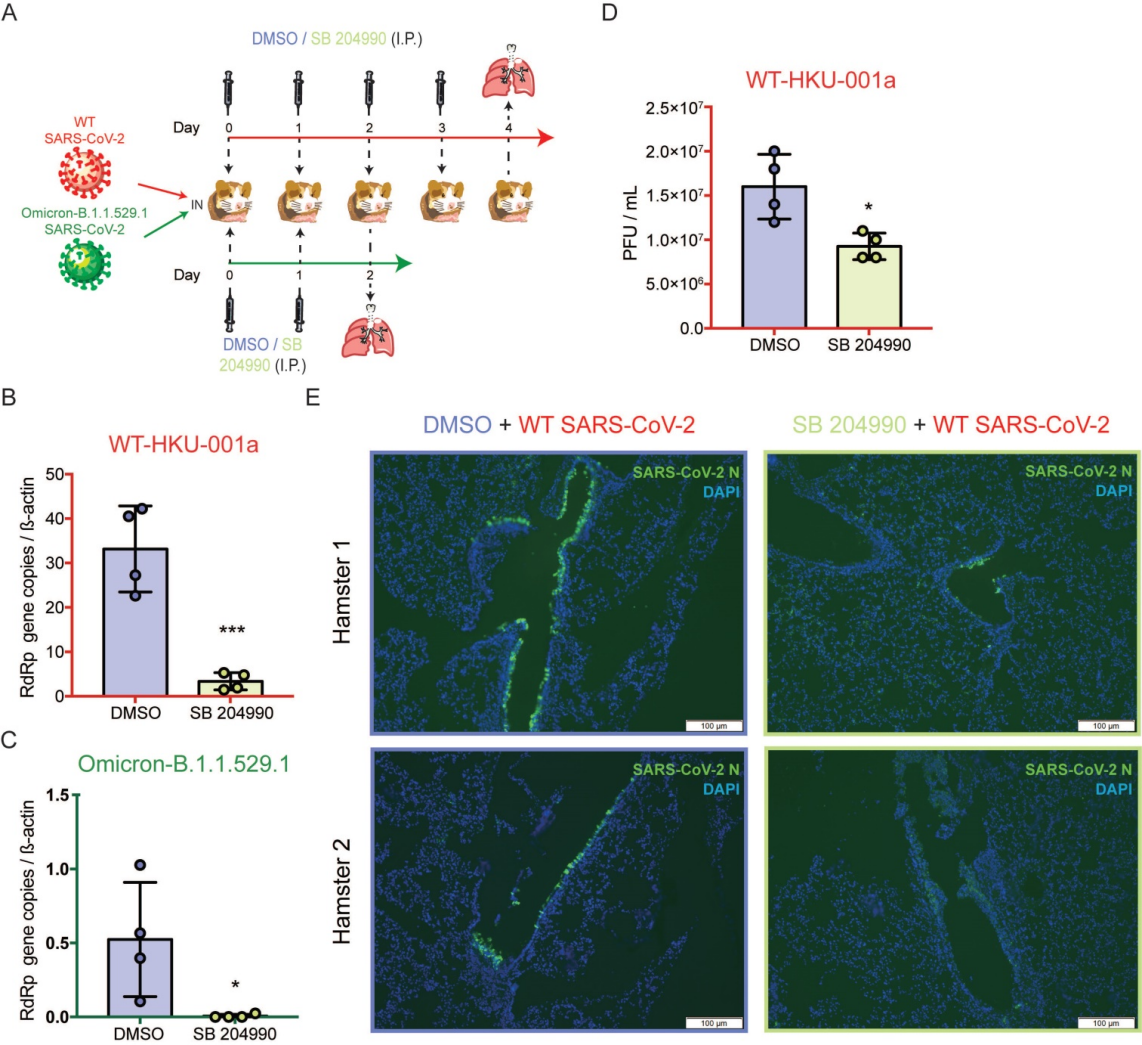
SB 204990 suppressed WT and Omicron BA.1 SARS-CoV-2 replications in a golden Syrian hamster model. Hamsters were intranasally inoculated with 3×10^3^ PFU per hamster of SARS-CoV-2 WT or Omicron BA.1. At 6 hours post-infection each hamster was treated intraperitoneally with 10mg/kg of SB 204990 or 5% DMSO at a final volume of 1000 µL PBS. The infected hamsters were subsequently treated with SB 204990 or DMSO at 1, 2, and 3 days post infection for a total of 2 or 4 doses. The infected hamsters were sacrificed on day 2 (Omicron-infected hamsters) or day 4 post-infection (SARS-CoV-2 WT-infected) for virological and histological assessments. (A) Schematic illustration of the *in vivo* study. (B) Viral RdRp gene copies in hamster lung tissues that were infected by SARS-CoV-2 WT were quantified by qRT-PCR. (C) Viral RdRp gene copies in hamster lung tissues that were infected by Omicron BA.1 were quantified by qRT-PCR. (D) Infectious titer of SARS-CoV-2 WT in hamster lung tissues were quantified by plaque assays. (E) Viral antigen in hamster lung tissues from SARS-CoV-2 WT-infected hamsters were detected with an in-house anti-SARS-CoV-2 nucleocapsid protein antibody. Cell nuclei were identified with DAPI stain. Data represented mean and standard deviation from two independent experiments. Statistical differences were considered significant when *P* < 0.05. *P < 0.05 and ***P < 0.001. Scale bar in (E) represented 100µm.

To further investigate the physiological role of ACLY *in vivo*, we next evaluated the outcome of ACLY inhibition using our established golden Syrian hamster model as previously described with slight modifications 47, 48. The hamsters were intranasally challenged with SARS-CoV-2 WT or Omicron at 3×10^3^ PFU and were intraperitoneally treated with SB 204990 or DMSO for 2 or 4 days. The infected hamsters were sacrificed at 2 or 4 days post-infection and their lung tissues were collected for viral gene copy and/or infectious titer quantification (**Figure 7A**). Consistent with our *in vitro* data, the *in vivo* data showed that the selective ACLY inhibitor SB 204990 significantly diminished the amount of viral RdRp gene copies in hamster lungs by about 90.0% (*P* = 0.0009) for WT (**Figure 7B**) and 98.8% (*P* = 0.0365) for Omicron (**Figure 7C**), respectively, when compared to the DMSO-treated groups. In keeping with the viral gene copy result, SB 204990 significantly reduced the amount of live infectious viral particles by 57.8% (*P* = 0.0141) in the lungs of SARS-CoV-2 WT-infected hamsters comparing to the DMSO-treated group (**Figure 7D**). To further evaluate the impact of ACLY on SARS-CoV-2 antigen expression in hamster lungs, we performed immunofluorescence staining for the lungs of SARS-CoV-2 WT-infected hamsters treated with SB 204990 or DMSO. Our results showed that the amount of detectable SARS-CoV-2 nucleocapsid protein was substantially lower in the SB 204990-treated hamsters than the DMSO-treated hamsters (**Figure 7E**). Taken together, these results indicated that inhibition of ACLY by SB 204990 effectively inhibits both SARS-CoV-2 WT and Omicron replication *in vivo*.

## Discussion

Viruses are known to perturb the host metabolome to facilitate their own replication. For example, RNA viruses such as rhinovirus 49, influenza virus 50, Zika virus 51, Dengue virus 52, hepatitis C virus 53, and human immunodeficiency virus 54 can hijack the TCA cycle during viral replication. Recent studies have established methods for studying the metabolomics of SARS-CoV-2 infection 55-58. However, detailed downstream analysis and the role of the identified host pathways during viral replication were not fully investigated. In this study, we investigated the SARS-CoV-2-induced metabolome perturbations in COVID-19 patient plasma samples. Utilizing combined data analysis from GC-MS-based targeted metabolomics and LC-MS-based untargeted metabolomics, we identified several metabolites participating in the TCA cycle that were significantly perturbed in COVID-19 patient samples. At the same time, a number of metabolites involved in biosynthesis of amino acid were downregulated in COVID-19 patient samples. The observed downregulated metabolites could be a result of the rapid SARS-CoV-2 replication, which used these metabolites (aspartic acid, tryptophan, lysine, asparagine, histidine, and glutamine) as building-blocks of viral synthesis.

Extending from the findings of a recent report on the association between SARS-CoV-2 infection and the host TCA cycle 59, in this study we further delineated the interaction between SARS-CoV-2 and specific host components in the TCA cycle, and identified ACLY as an important host factor involved in SARS-CoV-2 replication. ACLY is an essential metabolic enzyme that participates in fatty acid biosynthesis and is responsible for the synthesis of acetyl-CoA 46, which is then used in various critical biosynthetic pathways including lipogenesis and cholesterogenesis 46. Lipogenesis is the conversion of fatty acids and glycerol into fats, or metabolic reactions of which acetyl-CoA is converted to triglycerides for fat storages 60. Viruses rely on lipids for multiple stages of the viral replication cycle, including entry, formation of viral replication complex, replication, egress, and particle release 61. Viruses such as hepatitis C virus and human immunodeficiency virus are known to perturb lipogenesis for optimal replications 62, 63. MERS-CoV can reprogram the host lipid metabolism through the SREBPs-mediated lipogenesis to promote viral protein palmitoylation and formation of double-membrane vesicles for efficient viral replication 64. Hepatitis B virus interferes with the lipid metabolism through ACLY during viral replication 65. Therefore, ACLY inhibition may effectively disturb viral replication by reducing the amount of lipids that are crucial for various steps of the virus life cycle.

SARS-CoV-2 VOCs are associated with altered pathogenicity, transmissibility, and/or immune evasiveness 5. Most clinically approved monoclonal antibodies were found to be ineffective against the most recently emerging Omicron 6, 9-11. Specific inhibitors of key enzymes within the three mini pathways of the TCA cycle significantly reduced SARS-CoV-2 replication *in vitro* at non-toxic concentrations. Importantly, SB 204990 and Bempedoic acid also significantly inhibited the Delta and Omicron variants, suggesting that these host-targeting inhibitors are effective against emerging VOCs. Gene depletion using ACLY siRNA in Caco2 cells validated the findings of the selective small molecule inhibitors. Finally, we showed that SB 204990-treated Syrian hamsters exhibited significantly reduced viral burdens. Taken together, our findings identified ACLY as an important host factor that could be targeted to inhibit not only WT SARS-CoV-2, but also variants including Delta and Omicron.

## Figures and Tables

**Table 1:** The 21 metabolites (GC-MS analysis) that were significantly different between COVID-19 patients and non-infected volunteers

ID	Metabolite name	Fold change of COVID-19 vs non-infected	P-value	VIP	Confirmation	KEGG-ID	HMDB-ID	Involved pathway
GC-1	2-Hydroxyglutaric acid	1.73	6.83E-05	1.48	STD	C02630	HMDB0059655	Butanoate metabolism
GC-2	3-Hydroxybutyric acid	2.17	6.64E-03	1.4	STD	C01089	HMDB0000357	Butanoate metabolism
GC-3	Alpha-ketoglutaric acid	6.13	3.93E-07	1.78	STD	C00026	HMDB0000208	TCA cycle
GC-4	Pyruvic acid	3.08	8.20E-11	2.21	STD	C00022	HMDB0000243	TCA cycle
GC-5	Malic acid	1.97	8.96E-03	1.04	STD	C00149	HMDB0000744	TCA cycle
GC-6	Fumaric acid	1.59	5.96E-03	1.09	STD	C00122	HMDB0000134	TCA cycle
GC-7	Phosphoenolpyruvic acid	1.58	1.38E-02	0.99	MSMS	C00074	HMDB0000263	TCA cycle
GC-8	Succinic acid	1.57	4.19E-02	0.92	STD	C00042	HMDB0000254	TCA cycle
GC-9	Isocitric acid	0.64	2.60E-04	1.37	STD	C00311	HMDB0000193	TCA cycle
GC-10	Aspartic acid	0.66	3.28E-02	0.94	STD	C00049	HMDB0000191	Biosynthesis of amino acids
GC-11	Tryptophan	0.66	1.79E-04	1.49	MSMS	C00078	HMDB0000929	Biosynthesis of amino acids
GC-12	Lysine	0.65	7.72E-03	1.1	STD	C00047	HMDB0000182	Biosynthesis of amino acids
GC-13	Asparagine	0.63	9.74E-03	1.08	STD	C00152	HMDB0000168	Biosynthesis of amino acids
GC-14	Histidine	0.51	6.80E-05	1.6	MSMS	C00135	HMDB0000177	Biosynthesis of amino acids
GC-15	Glutamine	0.42	1.34E-04	1.4	MSMS	C00064	HMDB0000641	Biosynthesis of amino acids
GC-16	Homovanillic acid	0.34	5.54E-03	1.11	STD	C05582	HMDB0000118	Tyrosine metabolism
GC-17	5-hydroxyindole-3-acetic acid	0.28	1.97E-02	1.02	STD	C05635	HMDB0000763	Tryptophan metabolism
GC-18	6-Phosphogluconic acid	0.66	1.75E-03	1.32	MSMS	C00345	HMDB0001316	Pentose phosphate pathway
GC-19	Glucose	0.43	1.21E-07	1.9	STD	C00031	HMDB0000122	Galactose metabolism
GC-20	Mannose	0.39	1.28E-09	2.06	MSMS	C00159	HMDB0000169	Galactose metabolism
GC-21	Urea	0.6	1.03E-06	1.73	MSMS	C00086	HMDB0000294	Purine metabolism

**Table 2:** The 31 metabolites (LC-MS analysis) that were significantly different between COVID-19 patients and non-infected volunteers

		ID	Metabolite name	Fold change of patient vs healthy	Adjust P-value	VIP	Confirmation	KEGG-ID	HMDB-ID	Involved pathway
	1	74	3-Hydroxyanthranilic acid	0.24	0.0030	<1	MSMS	C00632	HMDB0001476	Amino acid and metabolism
	2	199	Citrulline	0.62	0.0026	<1	MSMS	C00327	HMDB0000904	Amino acid and metabolism
	3	18	Creatinine	0.54	0.0165	1.15	MSMS	C00791	HMDB0000562	Amino acid and metabolism
	4	92	3-Methylhistidine	0.24	0.0049	<1	MSMS	C01152	HMDB0000479	Amino acid and metabolism
**	**5**	**251**	**Piperine**	**0.24**	**0.0049**	**1.78**	**MSMS**	**C03882**	**HMDB0029377**	**Amino acid derivative**
	6	122	3,4-Dihydroxybenzoic acid	0.34	0.0001	1.33	MSMS	C00230	HMDB0001856	Biosynthesis of secondary metabolites
*	**7**	**133**	**Caffeine**	**0.32**	**0.0401**	**1.54**	**MSMS**	**C07481**	**HMDB0001847**	**Caffeine metabolism**
	8	276	1,7-Dimethyluric acid	0.48	0.0408	<1	MSMS	C16356	HMDB0011103	Caffeine metabolism
	9	551	PC(O-16:0/0:0)	0.62	0.0423	2.05	MSMS	C04317	NA	Ether lipid metabolism
	10	887	PC(O-36:5)	0.64	0.0000	1.84	MSMS	C05212	HMDB0013415	Ether lipid metabolism
	11	866	PC(P-34:2)	0.60	0.0001	1.19	MSMS	C05212	HMDB0008029	Ether lipid metabolism
***	**12**	**815**	**PE(O-16:0/18:3)**	**0.39**	**0.0000**	**<1**	**MSMS**	**C04475**	**NA**	**Ether lipid metabolism**
	13	872	PE(O-16:0/22:6)	0.63	0.0071	1.05	MSMS	C04475	NA	Ether lipid metabolism
	14	844	PE(O-36:4)	0.51	0.0005	<1	MSMS	C04475	NA	Ether lipid metabolism
	15	491	PE(P-16:0/0:0)	0.44	0.0059	<1	MSMS	C04635	HMDB0011152	Ether lipid metabolism
	16	523	PE(P-18:0/0:0)	0.40	0.0066	<1	MSMS	C04635	HMDB0240598	Ether lipid metabolism
	17	637	PC(20:5/0:0)	0.63	0.0258	1.12	MSMS	C04230	HMDB0010397	Glycerophospholipid metabolism
	18	863	PE(36:4)	1.61	0.0016	<1	MSMS	C00350	HMDB0008844	Glycerophospholipid metabolism
	19	32	Nicotinamide	1.92	0.0018	<1	MSMS	C00153	HMDB0001406	Nicotinate and nicotinamide metabolism
**	**20**	**53**	**Trigonelline**	**0.08**	**0.0084**	**1.53**	**MSMS**	**C01004**	**HMDB0000875**	**Nicotinate and nicotinamide metabolism**
	21	241	1-Methyladenosine	0.56	0.0267	<1	MSMS	C02494	HMDB0003331	No pathway
	22	451	CMPF	0.52	0.0007	2.98	MSMS	NA	HMDB0061112	No pathway
	23	317	Indolelactic acid	0.45	0.0375	<1	MSMS	C02043	HMDB0000671	No pathway
	24	342	Indoxyl sulfate	0.28	0.0018	7.37	MSMS	NA	HMDB0000682	No pathway
	25	23	3-Hydroxyvaleric acid	1.51	0.0278	<1	MSMS	NA	HMDB0000531	No pathway
**	**26**	**588**	**Inosine**	**0.09**	**0.0053**	**2.37**	**MSMS**	**C00294**	**HMDB0000195**	**Purine metabolism**
	27	474	Pseudouridine	0.33	0.0208	<1	MSMS	C02067	HMDB0000767	Pyrimidine metabolism
	28	472	O-oleoylcarnitine	1.75	0.0006	2.80	STD	C02301	HMDB0005065	Thermogenesis
	29	431	O-palmitoleoylcarnitine	2.17	0.0003	<1	STD	C02301	HMDB0013207	Thermogenesis
	30	758	Eicosenoic acid	1.70	0.0012	<1	MSMS	C16526	HMDB0002231	Unsaturated fatty acids
	31	232	Stearidonic Acid	0.63	0.0027	<1	MSMS	C16300	HMDB0006547	Unsaturated fatty acids

The 'O-' prefix is used to indicate the presence of an alkyl ether substituent, e.g., PC (O-16:0/0:0), whereas the 'P-' prefix is used for the 1Z-alkenyl ether (Plasmalogen) substituent, e.g., PE (P-16:0/0:0). Abbreviation: PC, glycerophosphocholine; PE, glycerophosphoethanolamine; EtherLPC, ether-linked lysophosphatidylcholine; EtherLPE, ether-linked lysophosphatidylethanolamine; EtherPC, ether-linked phosphatidylcholine EtherPE, ether-linked phosphatidylethanolamine; NA, not available.
